# Perirenal fat thickness measured with computed tomography is a reliable estimate of perirenal fat mass

**DOI:** 10.1371/journal.pone.0175561

**Published:** 2017-04-19

**Authors:** Guillaume Favre, Caroline Grangeon-Chapon, Charles Raffaelli, Florence François-Chalmin, Antonio Iannelli, Vincent Esnault

**Affiliations:** 1 Nephrology Unit, Nice University Hospital, Nice, France; 2 University of Nice Sophia-Antipolis, Nice, France; 3 Institute for Research on Cancer and Aging, Nice (IRCAN), “Aging and Diabetes” team, Nice, France; 4 Nuclear Medicine Department, Nice University Hospital, Nice, France; 5 Radiology Department, Nice University Hospital, Nice, France; 6 Digestive Unit, Archet 2 Hospital, University Hospital of Nice, Nice, France; 7 Inserm, U1065, Team 8 “Hepatic complications of obesity”, Nice, France; INIA, SPAIN

## Abstract

Deposition of perirenal adipose tissue has been associated with adverse renal and cardiovascular events. We compared various methods to measure perirenal adipose tissue using computerized tomography (CT)-scan and performed correlations with anthropometric measures associated with renal and cardiovascular events. Voluntary overweight and obese subjects undergoing a CT-scan for diagnostic purposes were included in the study. Perirenal adipose tissue volume, adipose tissue area of the renal sinus and perirenal fat thickness were manually measured bilaterally. The intra- and inter-observer coefficient correlations and the correlation between the diverse measures of renal adipose tissue, subcutaneous (SC-)fat and anthropometrics measures were analyzed using Pearson's correlation tests. The forty included patients (24 men, 16 women) had a mean age of 57.6 ± 18.1 years and a mean body mass index of 28.9 ± 2.9 kg/m^2^. Despite comparable waist circumference, women had a greater SC-fat thickness compared to men, and therefore a smaller amount of visceral fat, as well as smaller perirenal fat volumes. Perirenal fat thickness was better correlated with perirenal fat volume than adipose area of the renal sinus (*p* <0.02). The adipose area of the renal sinus did not correlate with any anthropometric measures. In women, perirenal fat volume and thickness showed a negative correlation with SC-fat thickness and no correlation with waist circumference. In men, perirenal fat volume and thickness showed a positive correlation with waist circumference and no correlation with subcutaneous fat thickness. In conclusion, perirenal fat thickness measured with CT-scan at the level of the renal veins is a simple and reliable estimate of perirenal fat volume, that correlated negatively with SC-fat in women and positively with waist circumference in men. The adipose area of the renal sinus did not correlate with any anthropometric measure.

## Introduction

Perirenal adipose tissue fills the space outlined by the inner and outer boundaries of the kidney and renal fascia. This adipose depot also involves the renal sinus and the renal hilum. In mammals, perirenal adipose tissue is separated from renal parenchyma by a fibrous capsule [1] and its vascularization comes from branches of the abdominal aorta, which also supplies blood to the kidney cortex through the plexus of Turner [2], with potential vicious paracrine effects. The perirenal adipose tissue might be a component of visceral fat, mainly composed of white adipose cells that store energy and produce soluble pro-inflammatory adipocytokines. Recently, renal adipose tissue has been specifically linked to impaired kidney function and blood hypertension, independently from other adipose depots and body-mass index (BMI) [3, 4]. The surface area of fat from the renal sinus, measured by computerized tomography (CT)-scan [3] and magnetic resonance imaging (MRI) [4], was indeed associated with renal and cardiovascular adverse events. However, the possible deleterious effect of perirenal fat may rather depend on local production of adipocytokines directly acting in the kidney than on the expansion of fat in the renal sinus. Because the relationship between fat in the renal sinus and the whole mass of perirenal fat is unknown, the purpose of our study is to question the link between perirenal fat volume and fat area of the renal sinus.

CT-scan is an accurate tool to quantify adipose tissue depots. The density of adipose tissue in Hounsfield Unit (HU) is used to distinguish it from other tissues. This approach has been validated by direct measurement of fat tissue in humans [5]. Manual tracing of adipose boundaries with a handball cursor [6] appears to be more precise than automatized delimitation based on thresh-holding [7]. However, fat density (in HU) is highly variable among subjects and depends on the CT image [8].

We measured the various compartments of perirenal fat and investigated their correlations with anthropometric measures, which are known to be correlated with renal and cardiovascular adverse events. This study is the prerequisite for subsequent epidemiological studies to search for better correlations between perirenal fat and renal endpoints such as impaired glomerular filtration rate, albuminuria, arterial hypertension and kidney stones.

## Subjects and methods

Selection of patients: Forty patients undergoing a CT-scan for diagnostic purposes were prospectively selected to enter the study. The participants were included between november 2012 and july 2013 in the radiology department. The inclusion criteria were: age >18 years, BMI >25 kg/m^2^, CT-scan done for any purpose with 0.625-mm-thick slices, covering the area between the pubic symphysis and the 10^th^ thoracic vertebra. All patients with renal-structure abnormalities on the CT scan, including tumours and cysts, were excluded. The study was approved by the local ethics committee (Comité de Protection des Personnes Sud-Méditérannée) and promoted by the University Hospital of Nice. All patients gave their written informed consent to be included in this study.

Measurements: Anthropometric measurements included body height, measured with a wall-mounted stadiometer; body weight, measured with a digital electronic scale (SECA, Birmingham, UK); and waist circumference, measured in a standing position at the level of the iliac crests. All CT-scans were performed on Light Speed VCT 64 (General Electric, Milwaukee, USA) in a supine position. Data were reconstructed with Advantage Windows 4.4 software (GE, Milwaukee, USA) to obtain 10-mm-thick consecutive slices. Perirenal fat was separated from other tissues according to its density (in HU): the window centre was set at -120 HU and window-width ranged from –195 to –45 HU for further analyses. The limits of each compartment were drawn with a hand-controlled trackball cursor.

Perirenal fat was quantified in three separate ways. (1) Adipose area of the renal sinus was delimited by a tangent line touching the outer limits of the kidney and crossing over the renal hilum. The largest area was chosen as previously described. (2) Perirenal fat was delimited by tracing the boundaries of the kidney, the aforementioned tangent line and the perirenal fascia. The consecutive areas of perirenal fat were multiplied by the thickness of the slice and added to each other to calculate the perirenal fat volume. (3) Perirenal fat thickness was the maximal distance between the posterior wall of the kidney and the inner limit of the abdominal wall on a slice passing through the renal vein. All three aforementioned measurements were made on both sides for each patient.

Subcutaneous (SC)-fat thickness was defined as the largest distance between the skin and the outer limit of the muscular wall of the abdomen at the level of the umbilicus. Waist circumference was outlined manually and measured at this same level. All analyses were performed with blinded endpoint evaluation and separately by two observers to estimate inter-observer agreement. Each observer repeated the measures to determine the intra-observer variability using two anonymized copies of the CT-scans, numbered from 1–40 in random order.

Statistical analyses: Intra- and inter-observer variabilities were assessed by intra- and inter-class correlation coefficients (ICC). Calculations were made for both observers and for both sides in each patient. In consideration of the difference in adipose-tissue distribution between men and women, the mean values of each parameter were calculated separately for men and women. As the data were distributed normally, results were expressed as means ± standard deviations and comparisons were performed with Student's t-test.

Correlations between different measures of perirenal fat were searched for. Correlations between perirenal adipose tissue measurements and SC-fat thickness, BMI or waist circumferences (on CT-scan as well as clinical measures) were calculated separately for men and women. Pearson's correlation tests were used and the threshold for statistical significance was set at *p* <0.05.

## Results

### Study population

There were 24 (60%) men and 16 (40%) women with a mean age of 57.6 ± 18.1 years and a mean BMI of 28.9 ± 2.9 kg/m^2^ (Table 1).

**Table 1:** Main parameters.

	Women	Men
n	16	24
Age (years)	50.9 ± 5.2	61.0 ± 3.4
BMI (kg/m^2^)	29.9 ± 0.8	28.6 ± 0.6
Radiological waist circumference (cm)	111.1 ± 1.5	107.7 ± 1.2
Subcutaneous fat thickness (cm)	3.7 ± 0.2 *	2.6 ± 0.1
Perirenal fat volume (mL)	R: 212 ± 32 * L: 258 ± 37 *	R: 413 ± 33 L: 507 ± 39
Renal sinus fat area (mm^2^)	R: 234 ± 26 * L: 295 ± 26 *	R: 418 ± 36 L: 475 ± 42
Perirenal fat thickness (mm)	R: 9.8 ± 1.3 * L: 9.4 ± 1.4 *	R: 20.3 ± 1.3 L: 23.0 ± 1.8

R: right; L: left; BMI: body-mass index. Patients from both genders did not have significantly different BMIs or WCs. However, men displayed a statistically significant larger volume of perirenal fat and less subcutaneous fat.

* p<0.001

### Measurements of perirenal fat

**Fig 1:**
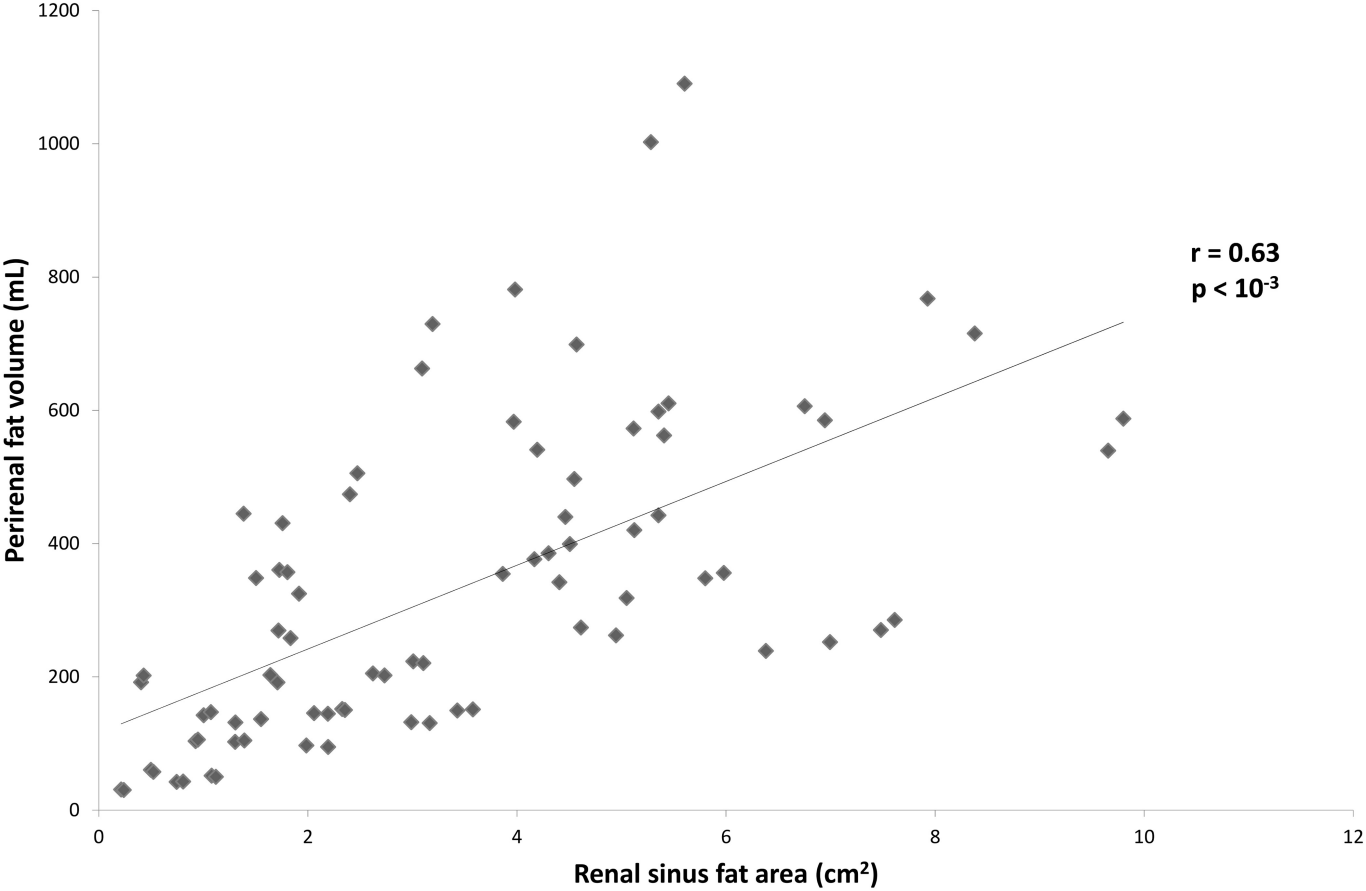
Correlations between volume of perirenal fat and area of sinus fat. Perirenal **fat** volume was positively correlated with **fat** area of the renal sinus in the whole population. Data are from both genders and from the right side of the body. Pearson's correlation test was performed.

**Fig 2:**
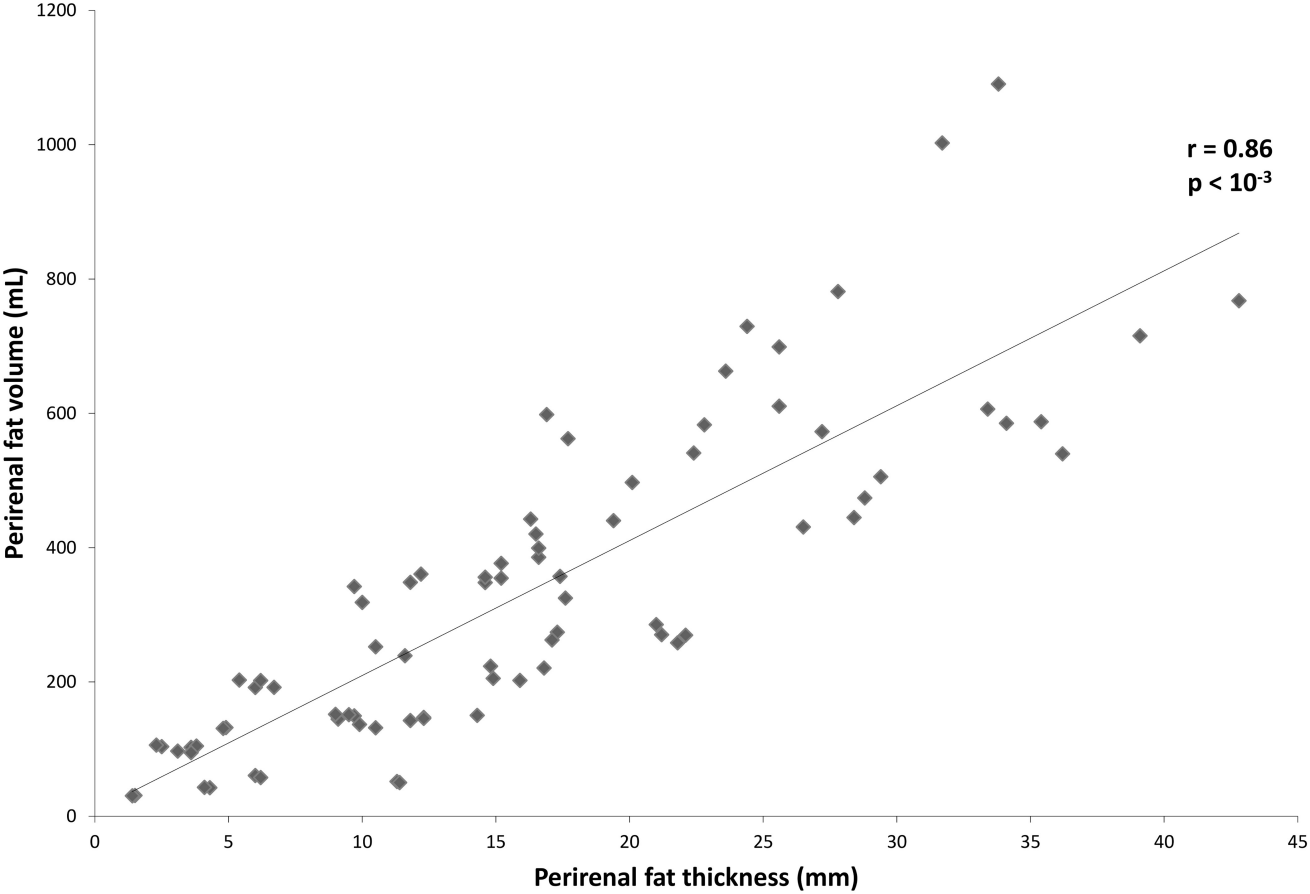
Correlations between volume and thickness of perirenal fat. Perirenal fat thickness was highly and positively correlated with perirenal **fat** volume. Data are from both genders and from the right side of the body. Pearson's correlation test was performed.

The perirenal fat volume on the left side was consistently greater compared to the right side in both genders. The intra- and inter-individual correlation coefficients (ICC) showed that measurements made in our study were reliable (Table 2). The perirenal adipose tissue volume was positively correlated with adipose area of the renal sinus in the whole population (r = 0.63 and 0.68, respectively, for the right and left side, *p*<0.001) (Fig 1). However, the perirenal fat thickness was better correlated with perirenal fat volume than adipose area of the renal sinus (*p* <0.02). Indeed, the perirenal fat thickness showed a strong positive correlation with perirenal fat volume (r = 0.86 and 0.91, respectively, for the right and left side, *p* <0.001) (Fig 2).

**Table 2:** Inter- and intra-observer correlation coefficients.

	Inter-observer variability			Intra-observer variability		
	Mean actual difference	Percentage difference (%)	ICC	Mean actual difference	Percentage difference (%)	ICC
Perirenal fat volume (mL)	R: 39 ± 49 / L: 47 ± 41	R: 12.1 ± 22.1 / L: 11.9 ± 15.2	0.97 / 0.98	**Observer D:**R:15 ± 12 / L: 17 ± 14	**Observer D:** R:4.9 ± 5.6 / L:4.7 ± 5.2	0.99 / 0.99
				**Observer Y:** R: 21 ± 23 / L: 25 ± 23	**Observer Y:** R:6.4 ± 9.6 / L:6.1 ± 8.5	0.99 /0.99
Renal sinus fat area (mm^2^)	R: 238.3 ± 137.5 / L: 244.0 ± 126.6	R: 51.4 ± 60.8 L: 46.6 ± 51.0	0.83 / 0.87	**Observer D:** R:51 ± 43 / L:57 ± 45	**Observer D:** R:8.8 ± 18.0 / L:8.9 ± 17.5	0.97 / 0.96
				**Observer Y:** R:21 ± 16 / L:26 ± 28	**Observer Y:** R:6.1 ± 7.1 / L:6.5 ±11.0	0.99 / 0.99
Perirenal fat thickness (mm)	R: 3.4 ± 2.9 / L: 2.1 ± 2.2	R: 22.2 ± 28.6/ L: 12.4 ± 17.6	0.93 / 0.97	**Observer D:** R:0.5 ± 0.5 / L:0.4 ± 0.4	**Observer D:** R:3.2 ± 4.4 / L:2.2 ± 2.3	0.99 / 0.99
				**Observer Y:** R:0.7 ± 0.9 / L:0.8 ± 0.9	**Observer Y:** R:4.3 ± 8.9 / L:4.8 ± 7.4	0.99 / 0.99
SC fat thickness (mm)	2.1 ± 2.4	6.9 ± 23.4	0.96	**Observer D:** 0.5 ± 0.4 / **Observer Y:** 1.0 ± 1.5	**Observer D:** 1.7 ± 4.2 / **Observer Y:** 3.4 ± 13.9	0.99 /0.99
WC (cm)	2.9 ± 2.4	2.7 ± 29.7	0.90	**Observer D:** 1.1 ± 0.8 / **Observer Y:** 1.2 ± 1.6	**Observer D:** 1.0 ± 10.0 / **Observer Y:** 1.1 ± 18.8	0.99 / 0.97

R: right side; L: left side; WC: waist circumference; SC: subcutaneous. For intra-observer variability, data are given arbitrarily for observer Y because they were similar to that from observer D. For inter-observer variability, data are arbitrarily given for the right side of the body because they were not significantly different from data from the left body side. An ICC close to 1 indicates a very good correlation.

### Differences between men and women

Women tended to be younger than men (50.9 ± 5.2 vs 61.0 ± 3.4 years, respectively, *p* = 0.1), but there were no significant differences in BMI and waist circumferences between men and women (Table 1). Despite comparable waist circumference, women had a greater SC-fat thickness compared to men (3.7 ± 0.2 vs 2.6 ± 0.1 cm, *p*<0.001), and therefore a smaller amount of visceral fat. Women had also smaller perirenal fat volumes compared to men, both on the right (212 ± 32 vs 413 ± 33 ml, *p*<0.001) and left side (258 ± 37 vs 507 ± 39 ml, *p*<0.001), smaller perirenal adipose tissue thickness both on the right (9.8 ± 1.3 vs 20.3 ± 1.3 mm, *p*<0.001) and left side (9.4 ± 1.4 vs 23.0 ± 1.8 mm, *p*<0.001), and smaller adipose area of the renal sinus both on the right (234 ± 26 vs 418 ± 36 mm^2^, *p*<0.001) and left side (295 ± 26 vs 475 ± 42 mm^2^, *p*<0.001).

### Correlation with anthropometric parameters

The associations between perirenal fat (volume, thickness, or adipose area of the renal sinus) and anthropometric measures (SC-fat thickness and waist circumference) were then investigated. Waist circumference was taken as a marker of total abdominal adipose tissue content (subcutaneous and visceral fat). The adipose area of the renal sinus did not correlate with any anthropometric measures. In women, perirenal adipose tissue volume as well as perirenal fat thickness showed a negative correlation with SC-fat thickness, and no significant correlation with waist circumference (Table 3). In men, perirenal adipose tissue volume as well as perirenal fat thickness showed a positive correlation with waist circumference, and no correlation with subcutaneous fat thickness (Table 3). Of note, BMI was not significantly correlated with any measure of perirenal fat.

**Table 3:** Correlations between perirenal fat volume and extra-renal fat deposits in both genders.

	Perirenal fat volume		Renal sinus fat area		Perirenal fat thickness	
Women	WC	SC fat thickness	WC	SC fat thickness	WC	SC fat thickness
Right side	0.21	-0.44 *	0.21	-0.29	0.20	-0.37 *
Left side	0.25	-0.45 ♮	0.24	-0.26	0.22	-0.51 ♮
	Perirenal fat volume		Renal sinus fat area		Perirenal fat thickness	
Men	WC	SC fat thickness	WC	SC fat thickness	WC	SC fat thickness
Right side	0.50 ♯	-0.25	0.27	-0.27	0.36 *	-0.19
Left side	0.45 ♮	-0.28	0.27	-0.13	0.38 ♮	-0.29 *

Extra-renal fat depots evaluated according to waist circumference (WC) and subcutaneous (SC) fat. There was a negative correlation between SC-fat thickness and perirenal fat volume / thickness in women, but not in men. There was a positive correlation between waist circumference and perirenal fat volume / thickness in men, but not in women.

*P*-values: * <0.05 ♮<0.01 # <0.001

## Discussion

This study provides evidence that perirenal adipose tissue volume correlates with adipose area of the renal sinus in overweight and obese subjects. This is in line with data obtained in rabbits fed a high caloric diet, in which perirenal fat and renal sinus adipose tissue developed synchronously [9]. However, perirenal fat volume and thickness but not the adipose area of the renal sinus showed correlations with anthropometric measures associated with renal and cardiovascular outcomes. Since the adipose area of the renal sinus has been associated with negative outcomes in epidemiological studies, we hypothetized that even stronger association might be found when studying the perirenal fat thickness.

The perirenal fat thickness, measured on CT-scan at the level of the renal vein showed a high correlation with perirenal adipose tissue volume. Therefore, it appears to be a good substitute for the time-consuming measurement of perirenal adipose tissue volume by 3D reconstruction. Furthermore, the intra- and inter-observer correlation coefficients of perirenal fat thickness were higher than those for the adipose area of the renal sinus, and therefore, the easy-to-measure perirenal fat thickness may be a good candidate for further epidemiological studies.

We confirm that perirenal fat may be a component of visceral fat, since its volume correlates with visceral fat but not with subcutaneous fat volumes. Women had lower perirenal fat and visceral fat volumes than men of comparable BMI and waist circumference. In contrast with waist circumference, BMI is known to be a poor marker of visceral fat volume and is also a poor marker of perirenal fat volume. Indeed, we found no correlation between BMI and perirenal fat. This is in agreement with data reported by Eisner et al, who found a weak correlation between BMI and perirenal fat in 123 patients [10]. In contrast, we found a significant and positive correlation between the volume of perirenal adipose tissues and waist circumference in men. This is consistent with the known associations between large adipose areas of the renal sinus and high intra-abdominal adipose tissue volume, as assessed by CT-scan [3] and MRI [4].

In diet-induced obesity in rodents, the perirenal fat is invaded by inflammatory macrophages and takes part into the production of adipocytokines together with other visceral fat deposits [11, 12]. The close vicinity and the common vascular link between perirenal fat and the kidney cortex through the plexus of Turner suggest that adipocytokines from perirenal fat could modify kidney function more directly than any other fat deposition. Indeed, it is remarkable that perirenal fat thickness, measured by ultrasonography in diabetic patients is negatively correlated with kidney function [13], and that the adipose area of the renal sinus is linked to impaired kidney function independently from the intra-abdominal fat in the Framingham cohort [3]. Moreover, the adipose area of the renal sinus is independently linked to hypertension [4]. Therefore, one might speculate that perirenal white adipose cells might produce adipocytokines resulting in kidney function loss and renal fibrosis [14]. Indeed, obsolescent glomeruli appear earlier in the outer cortex. Angiotensin II, TNF-alpha and leptin are synthetized by adipose tissue and the stimulation of angiotensin II type 1 receptors [15], and TNF-alpha and leptin receptors in the kidney triggers kidney fibrosis in rodents [16, 17]. Further, central obesity is associated to glomerular hyperfiltration [18] which is an independent factor of kidney function loss [19]. This raises the hypothesis that molecules produced by the perirenal adipose tissue could have a paracrine effect that can impair kidney function. Finally, the metabolic syndrome is associated with a urinary acidification defect leading to the formation of uric acid kidney stones [20–22]. It is possible that the production of ammoniac from proximal tubule cells in the kidney could be impaired by the toxicity of perirenal fat. This is currently being tested in a clinical trial (A0057-42).

The strength of our study relies in the use of a hand-controlled method to trace the boundaries of the different adipose compartments. This is the most accurate method because the automatized method of thresh-holding has been shown to be less precise than manual tracing of fat-tissue, owing to the variability of fat density in humans [8]. This accounts for the discrepancy between our measures and those reported by Foster et al. based on thresh-holding that found a smaller area of sinus adipose tissue. Indeed, Foster et al. reported median values of 0.97 (0.73, 1.34) cm^2^, whereas we found a mean of 3.44 ± 2.33 cm^2^ in our population (right side of body). This discrepancy is not likely to be a result of differences between subjects because the mean BMI reported by Foster et al. was 30.3 ± 5.7 kg/m^2^, which is comparable to the mean BMI we report herein (Table 1). However, the present study has several limitations. SC-fat thickness at the level of the umbilicus was used to estimate SC adipose mass [23] and variations of fat distribution along the vertical axis of the body may limit the accuracy of this measure. Since men tended to be older than women in this study, the gender-related associations between perirenal fat and extra-renal fat must be interpreted with caution. The correlations shall not be extended to normal weight or severe obese patients, who were not included in the study. Finally, we did not measure kidney function or blood pressure because the small number of our patients precluded the possibility to make any correlation with the outcome parameters.

In conclusion, perirenal fat may be a component of visceral fat that can be easily measured by perirenal fat thickness. Men exhibited larger volume of visceral and perirenal fat compared with women of comparable waist circumference. The adipose area of the renal sinus did not correlate with any anthropometric measures. In men, perirenal fat volume and thickness were positively correlated with waist circumference but not with BMI. In women, perirenal fat volume and thickness showed a negative correlation with SC-fat thickness. Further studies are now needed to correlate perirenal fat thickness with renal endpoints, including renal fibrosis and kidney stones.

## Supporting information

S1 Table Data basis including all measures. All measures were performed independently by 2 skilled observers. They are presented in details.
(DOCX)
