# Supplementary figures and images for: Perirenal fat thickness measured with computed tomography is a reliable estimate of perirenal fat mass
Source: PLoS One. 2017 Apr 19;12(4):e0175561. doi: 10.1371/journal.pone.0175561 (PMC5396915; doi:10.1371/journal.pone.0175561)

S1 Table: Data basis including all measures


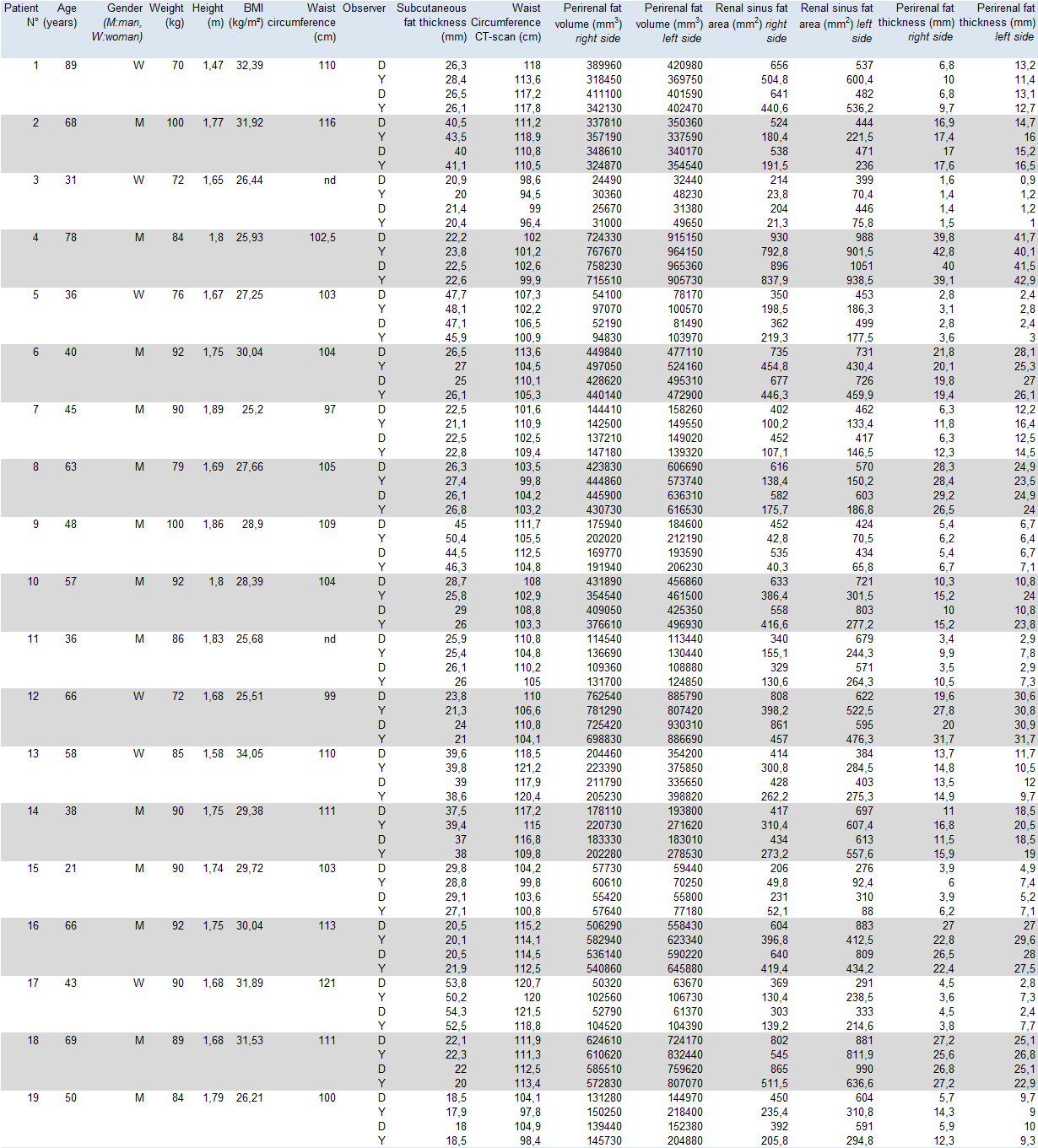


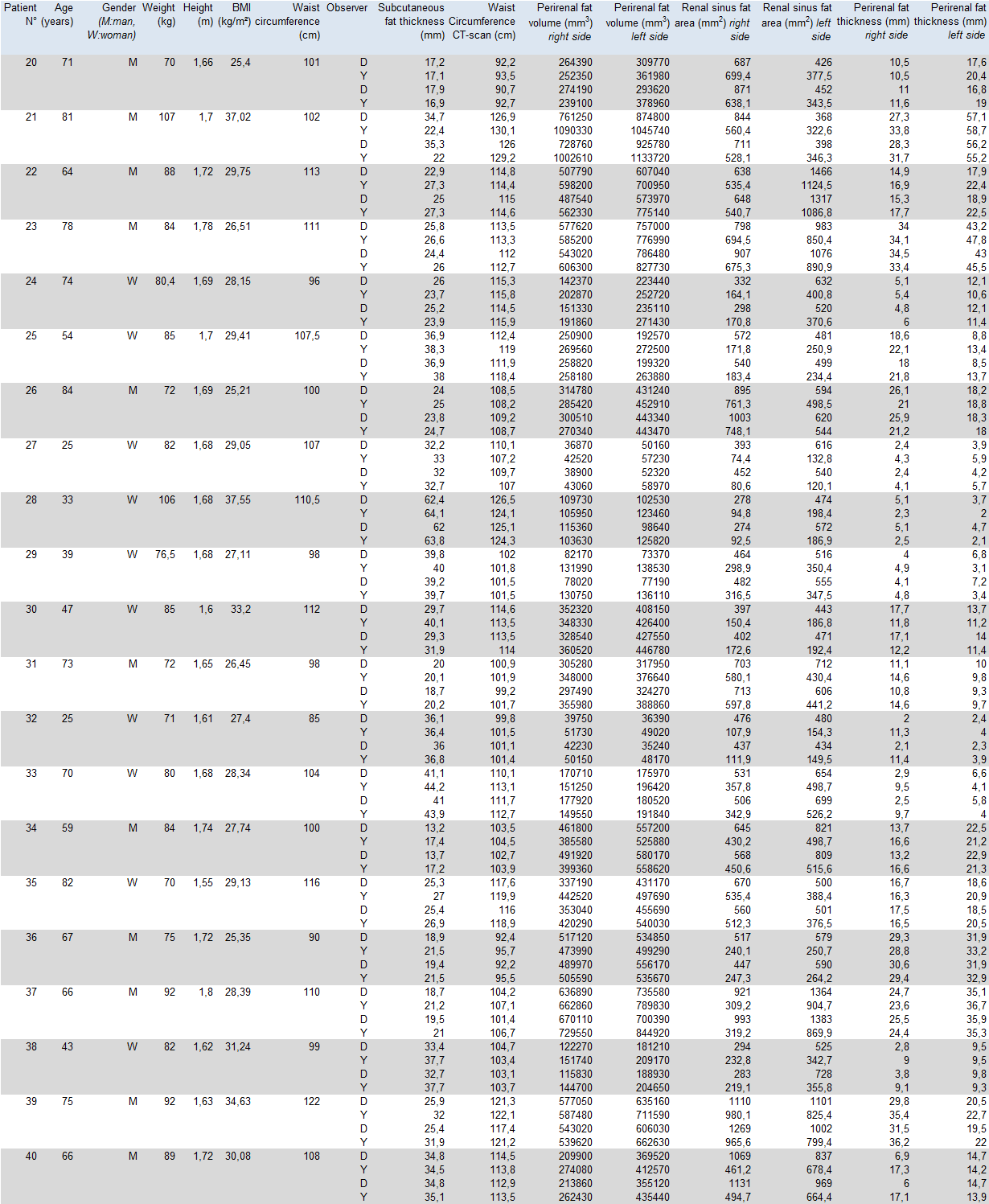

Supplement: S1 Table — All measures were performed independently by 2 skilled observers. They are presented in details. (DOCX) [file pone.0175561.s001.docx]
